# Ergothioneine attenuates psoriasis symptoms through modulation of M1/M2 macrophage polarisation via the NF-κB/JAK-STAT3 pathway

**DOI:** 10.3389/fphar.2025.1521743

**Published:** 2025-02-19

**Authors:** Ang Li, Yanjie Liu, Peiling Yu, Zhiyuan Zhang, Tengxiao Huang, Hang Li, Songzhi Wu, Xiaoyu Rong, Wensheng Liao, Hongqiang Wang, Yanzheng Gao

**Affiliations:** ^1^ Department of Orthopedics, Henan Provincial People’s Hospital, People’s Hospital of Zhengzhou University, Zhengzhou, Henan, China; ^2^ Department of Rehabilitation, Henan Second Provincial People’s Hospital, Xinzheng, Henan, China; ^3^ Department of Pathology, School of Basic Medical Sciences, Shandong University, Jinan, Shandong, China; ^4^ Department of Pathology, Qilu Hospital, Cheeloo College of Medicine, Shandong University, Jinan, Shandong, China; ^5^ Department of Orthopedics, Qilu Hospital, Cheeloo College of Medicine, Shandong University, Jinan, Shandong, China

**Keywords:** psoriasis, ergothioneine, macrophage, anti-inflammatory, NF-κB, Jak-STAT3

## Abstract

**Introduction:**

The underlying cause of psoriasis, a chronic inflammatory skin condition driven by an immune response, remains a topic of active investigation and is not yet fully elucidated. Recent studies have revealed that ergothioneine, a small molecule sulfur-containing histidine derivative that can be ingested from the daily diet and accumulated in the body, exhibits antioxidant capacity that is comparable to that of glutathione. Nevertheless, there is a paucity of empirical data concerning the precise impact of ergothioneine in the context of anti-inflammatory processes, particularly in the context of psoriasis. In the light of the aforementioned considerations, the present study was undertaken with the objective of conducting a comprehensive evaluation of the anti-inflammatory potential of ergothioneine (EGT) and to investigate its potential impact on the pathogenesis of psoriasis.

**Methods:**

The efficacy of EGT in reducing the extent of dorsal skin lesions in psoriasis model mice was confirmed through *in vivo* experimental observation. Furthermore, the inhibitory effect of EGT on inflammatory responses at the cellular level was investigated, specifically in LPS-induced mouse macrophage (RAW264.7) and human keratinocyte-forming cell (HaCaT) models.

**Results:**

The results demonstrated that the introduction of different concentrations of EGT into the LPS-induced inflammatory cell model resulted in notable anti-inflammatory effects, as evidenced by a reduction in inflammatory responses and a dose dependent decline in the concentrations of key inflammatory cytokines, including interleukin-1β (IL-1β), cyclooxygenase-2 (COX-2) and tumour necrosis factor-α (TNF-α). Furthermore, EGT was observed to reverse the LPS induced increase in the ratio of M1 to M2 macrophages. EGT was also observed to markedly suppress the LPS-induced phosphorylation of JAK/STAT3 and NF-κB, offering a novel insight into the anti-inflammatory mechanism of EGT.

**Discussion:**

In conclusion, the findings of the present study consistently demonstrated that ergothioneine had a significant ameliorative effect on the imiquimod-induced psoriasis model by modulating the NF-κB/JAK-STAT3 signalling pathway. This provides a strong experimental rationale for its potential application in psoriasis treatment.

## Graphical Abstract

**Figure:**
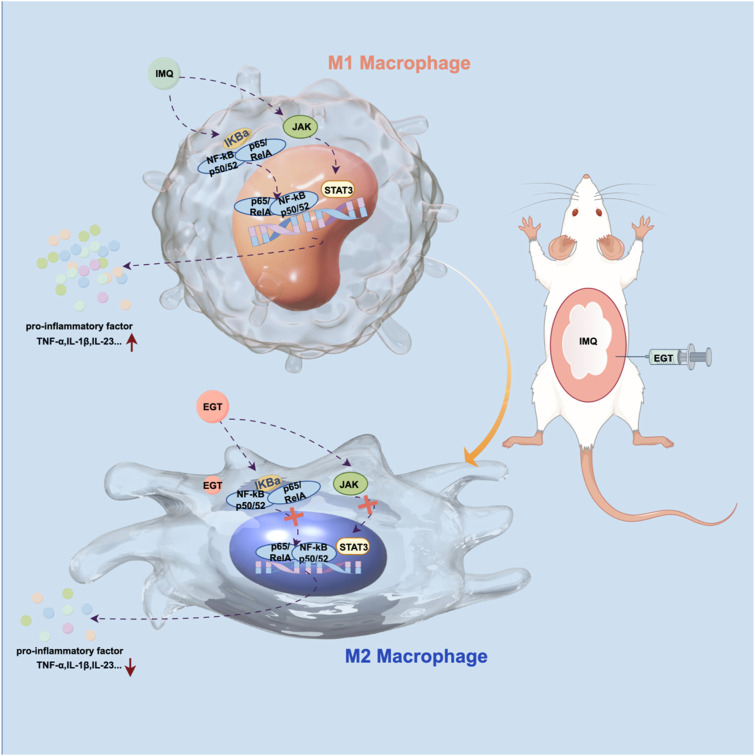
Mechanism of action of ergothioneine in reducing psoriasis symptoms Figure by (Figdraw).

## Introduction

The aetiology of psoriasis, a chronic and recurrent immune-mediated inflammatory skin disease that affects approximately 2%–3% of the global population (Greb et al., 2016), is widely accepted to involve a combination of genetic factors and external stimuli, including infections, psychological stress and specific medications (Armstrong and Read, 2020). The defining clinical features of the condition include erythematous lesions on the edges of the skin, accompanied by intense itching and silvery-white scales covering the skin surface (Rønholt and Iversen, 2017). Extensive research has demonstrated that macrophage polarisation can contribute to the development and progression of a range of inflammatory immune skin diseases, including psoriasis, AD, SLE and BD (Ross et al., 2021; van den Oord and de Wolf-Peeters, 1994; Buscher et al., 2017). In these diseases, the ratio of M1/M2-type macrophages is elevated, with M1-type macrophages playing a dominant role in perpetuating inflammatory responses and destructive cycles. Conversely, M2 macrophages are believed to exert an anti-inflammatory effect (Wang et al., 2022; Kobayashi et al., 2016; Shen et al., 2024).

The NF-κB pathway and the JAK/STAT3 pathway are classical cellular signaling pathways that have been identified to play a critical role in promoting cellular expression and accumulation of pro- and anti-inflammatory factors, as well as in activating and modulating inflammatory responses (Zhao et al., 2019; Wang C. et al., 2023; Li et al., 2024; Banerjee et al., 2017). A large body of evidence confirms that both pathways play a key role in the regulation and expression of cytokines in serum and damaged skin tissue of psoriasis patients (Kalliolias and Ivashkiv, 2016; Bell et al., 2003; Zhao et al., 2016; Pandey et al., 2023). In light of these findings, the NF-κB and JAK/STAT3 pathways are clearly a promising avenue for targeted therapy of psoriasis. Further studies are needed to develop safer and more effective therapeutic agents on this basis.

Ergothioneine (EGT) is a small molecule that is naturally synthesised by certain bacteria and fungi (Cheah and Halliwell, 2021; Marone et al., 2016). It belongs to a group of specific thiol derivatives that are easily absorbed by the body. It is important to note that this substance is not synthesised in animals or humans, but rather enters the body via the dietary route (Halliwell et al., 2023). To date, EGT has been demonstrated to possess a diverse range of biological functions, with its antioxidant and anti-inflammatory effects being particularly noteworthy (Chen et al., 2024). A multitude of studies have demonstrated that EGT possesses the capacity to impede the actions of TNF-α, expeditiously eliminate and curtail the generation of deleterious free radicals and inflammatory agents, and hinder the NF-κB signaling pathway and the JAK/STAT3 pathway to elicit anti-inflammatory effects. Furthermore, EGT has been shown to stimulate the intracellular antioxidant pathway through the p38 MAPK gene cascade (Zhao et al., 2019; Wang Z. et al., 2023; Duan et al., 2023; Jiang et al., 2024). The objective of this study was to examine the therapeutic efficacy of EGT in a psoriasis model and to elucidate the underlying mechanisms of action of EGT in reducing phosphorylated NF-κB and JAK/STAT3 expression. The findings of this study are anticipated to provide novel and effective targets and strategies for the clinical management of psoriasis.

## Materials and methods

### Mouse model of psoriasis induced by imiquimod

All animal care and experimental procedures in this study were conducted in strict accordance with the specifications established by the Shandong Provincial Laboratory Animal Management Committee and were formally approved by the Ethics Committee of Shandong University. The male BALB/c mice (7 weeks old, weighing between 15 and 20 g) used in the experiments were purchased from Vital River. The mice were housed in a specific pathogen-free environment with unrestricted access to food and water. The experimental design involved the random assignment of mice to five distinct groups (n = 6), comprising a blank control group, an EGT-treated group, an IMQ-treated group, an IMQ + NaCl-treated group, and an IMQ + EGT-treated group (70 mg/kg). To induce a psoriasis-like dermatitis, a 5% imiquimod cream was applied topically to the shaved backs of the mice at a dosage of 40 mg per day for a period of seven consecutive days. The treated group was administered a daily intraperitoneal injection of 70 mg/kg of EGT. During the course of the experiment, the body weights of the mice were measured on a daily basis, and photographs were taken using an iPad device to document their growth status. During day 7 of modelling, three experimenters performed PASI scores on the severity of psoriasis in the model mice. To ensure the objectivity and accuracy of the assessment results, all participants were blinded to the specific regimen of the drug treatment before data analysis, thus ensuring the fairness and reliability of the experimental results.

### Histological analysis and immunohistochemistry (IHC)

On the seventh day of the experiment, the following steps were taken to process and analyse the bare skin on the back of the mice. First, the samples were fixed in 4% paraformaldehyde and then embedded in paraffin. The embedded samples were then sectioned and stained with haematoxylin and eosin (H&E). For antigen retrieval, the sections were placed in sodium citrate solution at pH 6.0. The sections were then incubated with 3% hydrogen peroxide (H_2_O_2_) for 30 min at room temperature. The sections were then sealed with 5% goat serum for 2 h. After sealing, the sections were incubated with the appropriate primary antibodies at 4°C overnight, and the day after incubation, the sections were developed using a diaminobenzidine (DAB) substrate kit. Finally, the processed sections were imaged using a Nikon microscope. The antibodies used in this experiment are listed in Supplementary Table S2.

### PCR RNA extraction and real-time PCR

Mouse skin tissue or HaCaT cells were lysed in Trizol (Thermo Fisher Scientific) and RNA was isolated according to the manufacturer’s instructions. RNA was processed as cDNA using the TaqMan cDNA Synthesis Kit and SYBR Green mixture (Takara, Tokyo, Japan), with specific primer pairs as shown in Supplementary Table S1. Relative mRNA levels were calculated using β-actin as a reference control, and the respective mRNA expression was assessed using the 2^−ΔΔCT^ method.

### Western blot analysis

RAW264.7 cells, HaCaT cells and mouse skin tissue samples were harvested and subsequently lysed in a 1:100 buffer system containing PMSF (Beyotime Institute of Biotechnology) and RIPA (Beyotime Institute of Biotechnology). The resulting protein samples were denatured at high temperatures and, after effective separation on 10% SDS-PAGE (sodium dodecyl sulphate polyacrylamide gel electrophoresis) gels, transferred to PVDF membranes for subsequent analysis. For each experimental group, we measured the expression levels of several proteins, including iNOS (inducible nitric oxide synthase), COX-2 (cyclooxygenase-2), IL-1β (interleukin-1β), CD86, CD206, p-P65 and p-IκBα, using β-actin as a reference control. The antibodies used in this experiment are listed in Supplementary Table S2.

### Immunofluorescence staining

Cells (RAW264.7, HacaT) were inoculated into 24-well plates covered with glass slides and then treated with 100 ng/mL LPS and different concentration gradients (30, 60, 120 µM) of EGT for 24 h. At the end of the treatment, the cells were transferred to serum-free medium and incubated overnight. Cell monolayers on glass slides were then rinsed three times with PBS to remove residual treatment solution. The cells were fixed with 4% paraformaldehyde for 15 min at room temperature. The plasma membrane of the nucleus and the cells were then infiltrated with 0.1% Triton X-100 for 20 min to increase the permeability of the cell membrane. The treated cells were incubated with specific primary antibodies (CD86, CD206, anti-p65 and anti-iNOS antibodies) overnight at 4°C to ensure adequate binding. The following day, the appropriate rabbit-derived fluorescent secondary antibodies were used to bind to the primary antibodies and images of the treated cell sections were captured using an inverted fluorescence microscope (Zeiss, Germany). The antibodies used in this experiment are listed in Supplementary Table S2.

### Cell culture

Mouse monocyte-macrophage cell line (RAW264.7), human keratinocyte-forming cell line (HaCaT), and human monocyte (THP-1) were purchased from Cellverse (China). RAW264.7 cells and HaCaT cells were cultured in DMEM medium containing 10% FBS and 1% penicillin-streptomycin. THP-1 cells were cultured in RPMI1640 medium containing 10% FBS and 1% penicillin-streptomycin.

### Cell viability assay

Cell Counting Kit-8 (CCK8, Beyotime) was used to assess the viability of RAW264.7 and HaCaT cells in the presence or absence of LPS and different concentration gradients of EGT. 1*10^5 cells/well were inoculated into 96-well plates containing 100 μL cell culture medium per well and incubated for 24 h. Then 10 µL of CCK8 solution was added to each well and the incubation was continued for 1 h in an incubator at 37°C. At the end of the incubation, cell viability was assessed by measuring the absorbance of each well at 450 nm using an enzyme marker.

### Flow cytometry

For the *in vivo* assay, a single cell suspension was prepared by grinding the mouse spleen and rinsing the bone marrow from the mouse leg bone, adding erythrocyte lysis solution to completely lyse the erythrocytes in the suspension, and then FITC-conjugated anti-F4/80 and PE-conjugated anti-CD86 for M1 macrophage and FITC-conjugated anti-F4/80 and APC-conjugated anti-CD206 for M2 macrophage are added to the prepared cells and incubated for 1 h at 4°C under light protection.

For *in vitro* experiments, RAW264.7 cells were inoculated into six-well plates at a density of 2*10^5/well. These cells were then treated according to the established experimental protocol. At the end of the treatment, the appropriate antibodies were added to the resulting cell suspension. The F4/80 CD206 (M2) or F4/80 CD86 (M1) populations were determined by flow cytometry (BD FACSCalibur) and the results were analysed using FlowJo software (version 10).

### Network pharmacology

The GeneCards database (https://www.genecards.org), TTD (https://bidd.group/group/cjttd/) and OMIM(https://www.omim.org/)were used to search for psoriasis-related causative genes and a concatenation of these three databases was obtained. The chemical structure of ergothioneine was investigated using PibChem (https://pubchem.ncbi.nlm.nih.gov/) and the results were uploaded to the SwissTarget prediction (http://swisstargetprediction.ch/) database. Identification of potential target genes for ergothioneine using Venny analysis (https://bioinfogp.cnb.csic.es/tools/venny/index.html) was used to identify the overlap between psoriasis-associated disease-causing genes and ergothioneine target genes. These genes may play an important role in the alleviation of psoriasis symptoms by ergothioneine. The STRING (https://cn.string-db.org/) database was used to construct a protein-methionine interaction network with a feasibility setting of 0.4. The STRING database was used to construct a protein-methionine interaction network with a feasibility setting of 0.4. Each of the genes in this protein-methionine interaction network was analysed using the Cytohubb plug-in in Cytoscale software (version 3.8.1). gene significance.

### Data and the statistical analysis

Statistical analyses were performed in a blinded fashion to ensure the objectivity of the study. In this study, all experiments included a minimum sample size of n = 3 to ensure stability and reliability of results. We used GraphPad Prism 9 software for statistical analysis of the data, and all data collected were presented as mean ± SD. When statistically analysing differences between multiple groups, data were analysed using two statistical methods: one-way ANOVA or unpaired Student’s t-test. Statistical significance was considered when the P < 0.05. Post hoc tests were used only if F achieved P < 0.05, and there was no significant variance inhomogeneity.

## Results

### Roles of varying EGT contents in RAW264.7 cell and HaCaT cell activity

**FIGURE 1:**
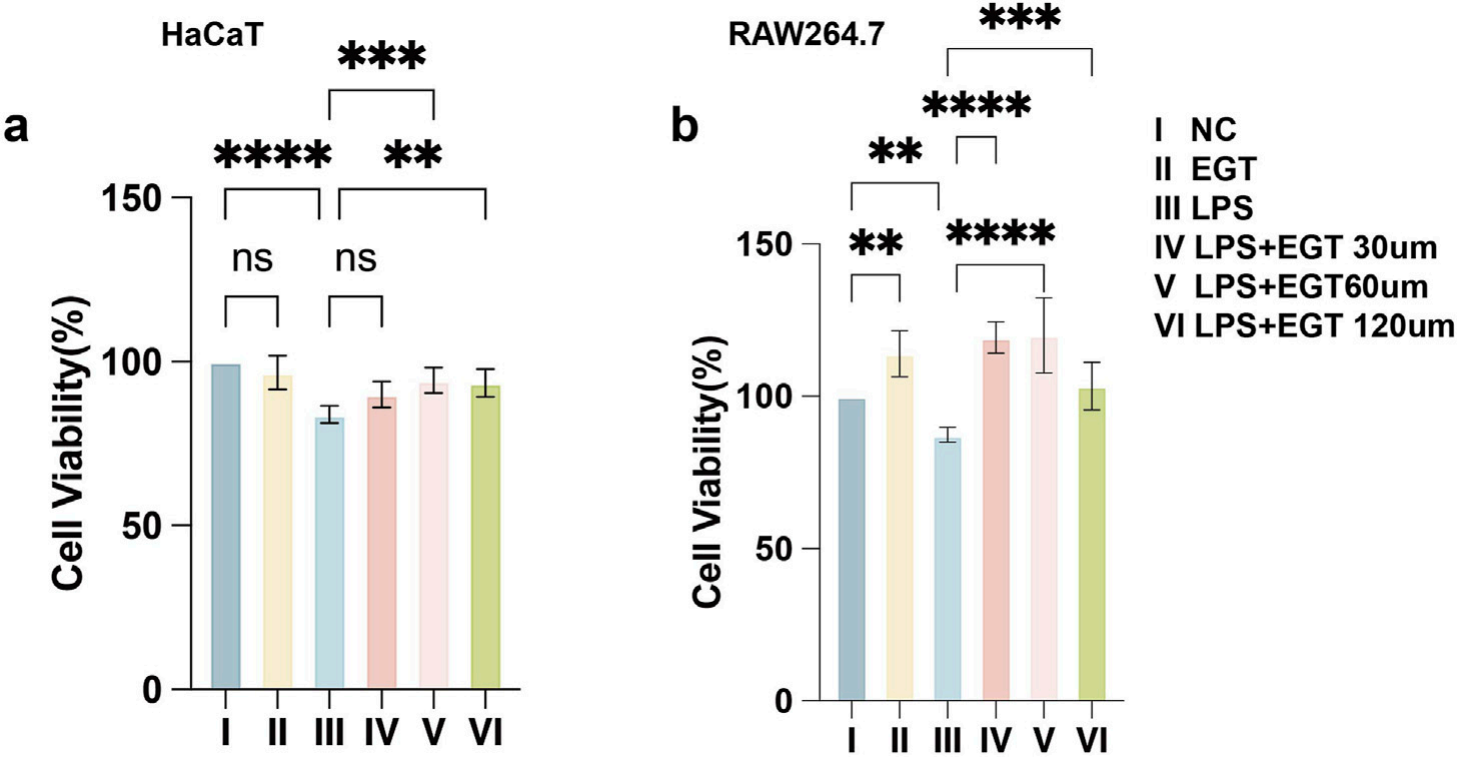
Effect of ergothioneine and LPS co-treatment on cell activity. **(A)** The effect of ergothioneine on HacaT cell activity was determined by CCK-8 assay. **(B)** Effect of ergothioneine on RAW264.7 cell activity determined by CCK-8 assay. Data are expressed as mean ± SD. *p < 0.05; p < 0.001; p < 0.0001.

We used the CCK-8 assay to determine the effects of different concentrations of EGT on the bioactivity of mouse macrophages RAW264.7 and human keratinocytes HaCaT. The experimental results showed that the addition of EGT alone at a concentration of 120 uM had no significant effect on the activity of the cells. When the cells were exposed to 100 ng/mL LPS, the cell activity decreased significantly. Notably, after further treatment of these cells with EGT at concentrations of 60, 120 µM for 24 h, we observed a significant increase in cell viability (Figures 1A, B). Based on these findings, we decided to conduct a more in-depth study using three concentrations of EGT (i.e., 30, 60, and 120 µM) in subsequent experiments.

### EGT modulates LPS-induced polarisation in M1/M2 macrophages

**FIGURE 2:**
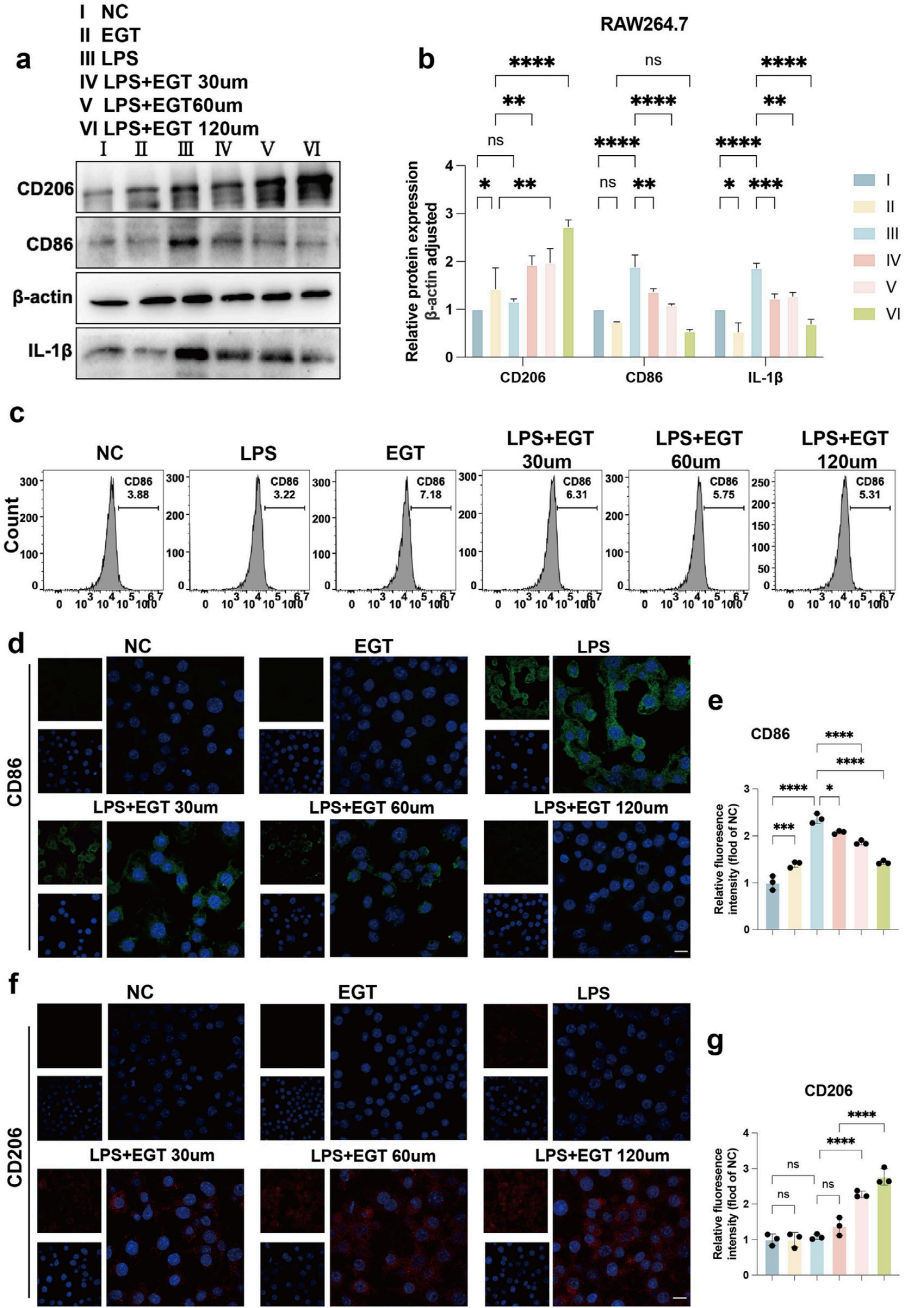
EGT regulates the ratio of M1 to M2 macrophages in RAW264.7 cells following the induction of these cells by LPS. **(A)** Protein expression levels of CD206 and CD86 in treated groups of RAW264.7 macrophages treated with LPS in the presence or absence of EGT for 24 h were detected by Western blot. **(B)** A statistical graph of protein quantification in **(A)**. **(C)** The proportion of CD86 M1 macrophages in each group, as detected by flow cytometry. **(D)** Cell immunofluorescence to detect the expression of CD86 in treated cells of each group. **(E)** Quantitative statistical plots of cell immunofluorescence. **(F)** Cell immunofluorescence to detect the expression of CD206 in treated cells of each group. **(G)** Quantitative statistical plots of cell immunofluorescence. Data are expressed as mean ± SD. *p < 0.05; p < 0.001; p < 0.0001.

We used mouse macrophages (RAW264.7) as a model to systematically assess the effects of EGT on macrophage polarisation processes. Specifically, when macrophages were exposed to LPS, their cells, which were originally in the M0 state, were transformed into M1-type macrophages. However, upon further addition of different concentrations of EGT, we observed that the proportion of M1-type macrophages showed a concentration-dependent decrease (Figures 2A, B). To verify the association of this phenomenon with EGT, we used flow cytometry for confirmation (Figure 2C). We then investigated the effect of EGT on the expression of macrophage polarisation markers. Using protein blotting and immunofluorescence techniques, we found that the expression of the M1-type macrophage marker CD86 showed a gradient decrease with increasing EGT concentration, while the expression of the M2-type macrophage marker CD206 increased significantly (Figures 2D–G). Similarly, we added 100 ng/mL phorbol 12-myristate 13-acetate (PMA) to human monocyte THP-1 cells to induce them into M0-type macrophages, which later went through the same treatment results as RAW264.7 cells, and also obtained results consistent with the above by Western blot (Supplementary Figures 1A, B). These results further confirm the important role of EGT in regulating macrophage polarisation.

### *In vitro* anti-inflammatory effects of EGT

**FIGURE 3:**
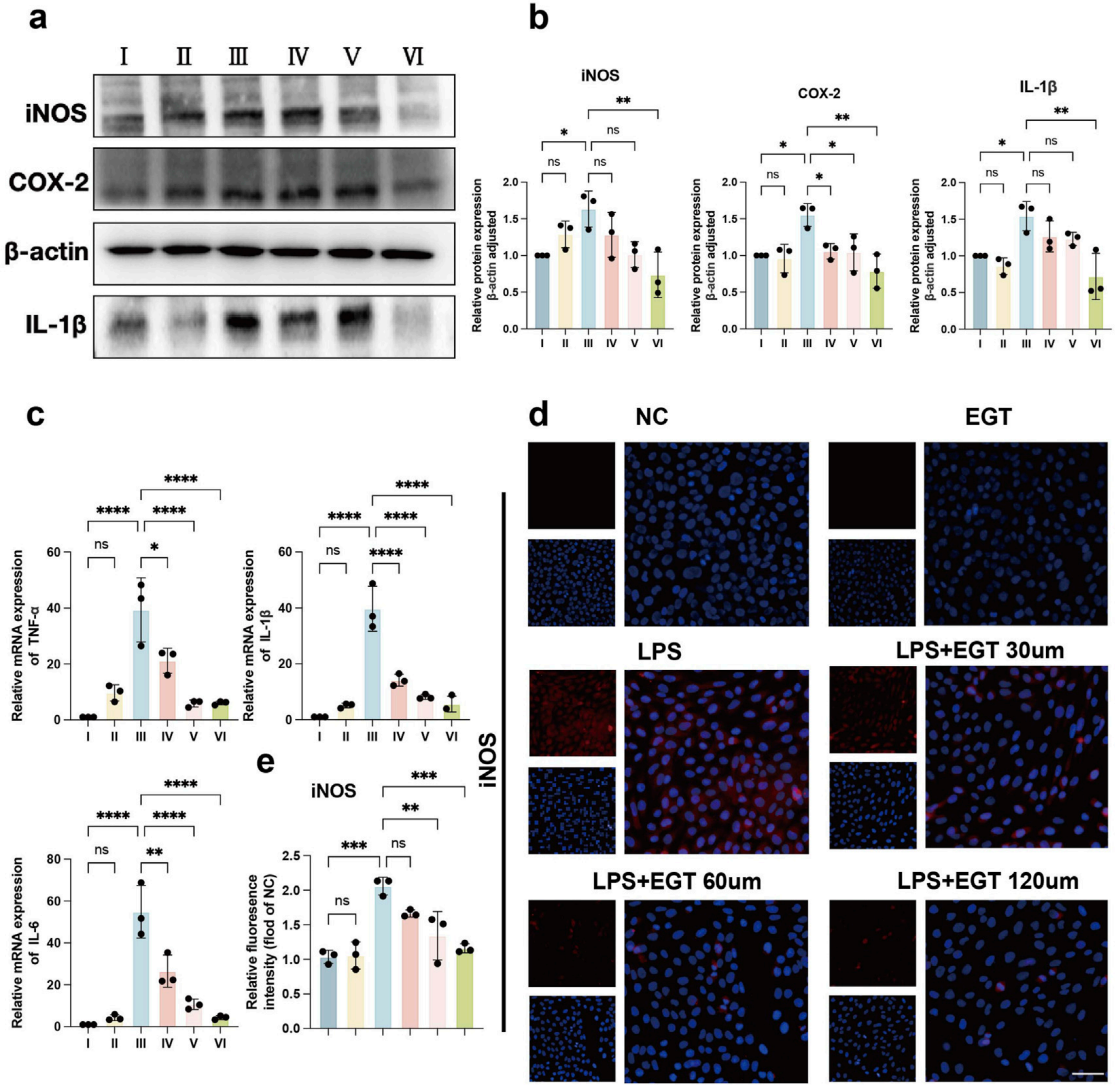
EGT has been demonstrated to possess anti-inflammatory effects in HacaT cells. **(A)** HacaT cells were treated with LPS in the presence or absence of EGT for 24 h. Protein expression of pro-inflammatory factors in each group was then quantified. **(B)** A statistical graph of protein quantification in **(A)** was generated. **(C)** mRNA expression of pro-inflammatory factors in treated cells was analysed by qRT-PCR. **(D)** iNOS expression in treated groups was detected by immunofluorescence. **(E)** A quantitative statistical graph of cell immunofluorescence was generated. Data are expressed as mean ± SD. *p < 0.05; p < 0.001; p < 0.0001.

To further investigate the anti-inflammatory effects of EGT in cells *in vitro*, we used LPS to simulate a cellular model of inflammation *in vitro*. The experimental results showed that when HaCaT cells were exposed to LPS stimulation, the expression levels of key inflammatory factors such as iNOS, COX-2 and IL-1β increased significantly. We then introduced different concentrations of EGT into these cells and observed that the expression of the above pro-inflammatory cytokines showed a significant decreasing trend with the gradual increase in EGT concentration (Figures 3A, B). Further analysis showed that at the mRNA level, the expression of pro-inflammatory factors such as TNF-α, IL-6 and IL-1β also showed a concentration-dependent decrease with the gradual increase in EGT concentration (Figure 3C). In addition, as visualised by immunofluorescence, the addition of EGT was able to significantly reduce the expression level of the pro-inflammatory factor iNOS (Figures 3D, E). The data suggest that the addition of EGT was able to demonstrate a significant concentration gradient anti-inflammatory effect in HaCaT keratinocytes.

### EGT reduces the severity of IMQ-induced psoriasis in mice. It also reduces the expression of associated pro-inflammatory factors

**FIGURE 4:**
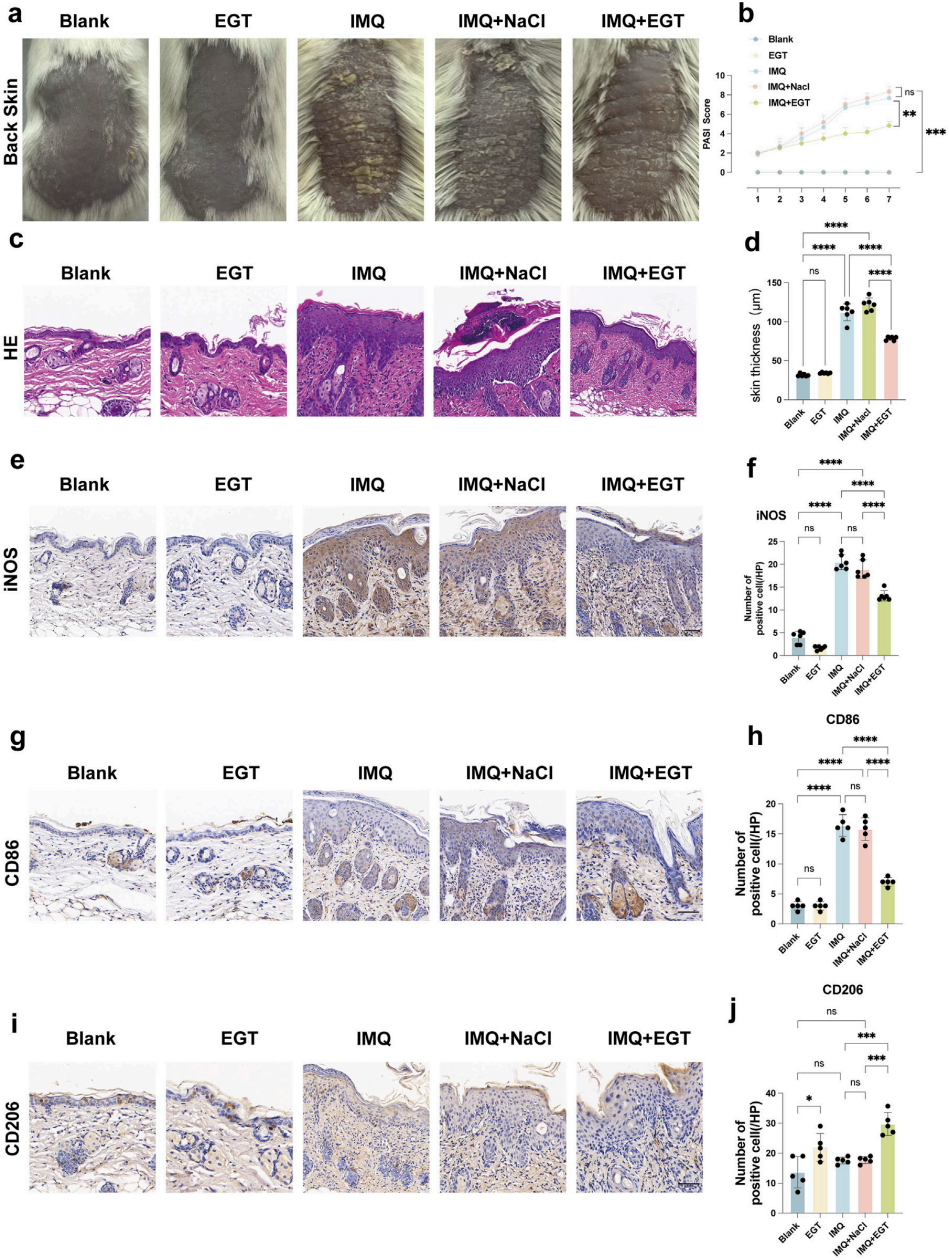
Ergothioneine attenuates IMQ-induced psoriasis-like skin symptoms. **(A)** Representative images of the dorsal skin of each group of mice on day 7. **(B)** PASI scores of mice with imiquimod-induced psoriasis for 7 consecutive days. **(C)** HE staining results (×200) of the dorsal skin of mice in each group. **(D)** Statistical graph of epidermal tissue thickness on the back of mice. **(E, F)** Immunohistochemistry (×200) and statistical graph of iNOS in the dorsal skin of mice. **(G, H)** IHC (×200) and positive cell counts of CD86 cells in dorsal skin tissues of mice in each group. **(I, J)** IHC (×200) and positive cell counts of CD206 cells in dorsal skin tissues of mice in each group, (scale bar = 100um). Data are expressed as mean ± SD. *p < 0.05; p < 0.001; p < 0.0001.

To verify the effect of EGT in the imiquimod-induced psoriasis mouse model, we used the following experimental approach: first, 5% imiquimod cream was applied to the shaved back skin of BALB/c mice to mimic the appearance of psoriasis-like lesions. Compared to the control group, which received no treatment, the dorsal skin of the mice in the model group showed significant pathological changes, as evidenced by typical psoriasis symptoms such as redness of the skin, production of large amounts of scales, a significant increase in skin thickness, and even the appearance of desquamation and crusting (Figures 4C, D). We then injected EGT (at a dose of 70 mg/kg) intraperitoneally into the mice in the treatment group to evaluate its therapeutic effect (Tang et al., 2018; Salama et al., 2021). The histopathologic HE results of spleen and kidney of mice at this therapeutic concentration showed no abnormalities (Supplementary Figure S2). The experimental results showed that the injection of EGT significantly alleviated the symptoms of the psoriasis mice. Compared with the model group, mice treated with EGT had a significant reduction in skin thickness and a significant reduction in scale production (Figure 4A). In addition, we performed a Psoriasis Area and Severity Index (PASI) score on the mice to further quantitatively assess the therapeutic effect of EGT. The results showed that the PASI scores of the mice in the EGT-treated group were significantly lower than those of the IMQ model group on the seventh day of the experiment (Figure 4B). In addition to the aforementioned improvement in skin symptoms, we also observed that the spleens of mice in the EGT-treated group showed a significant reduction compared to the model group (Supplementary Figure S3).

**FIGURE 5:**
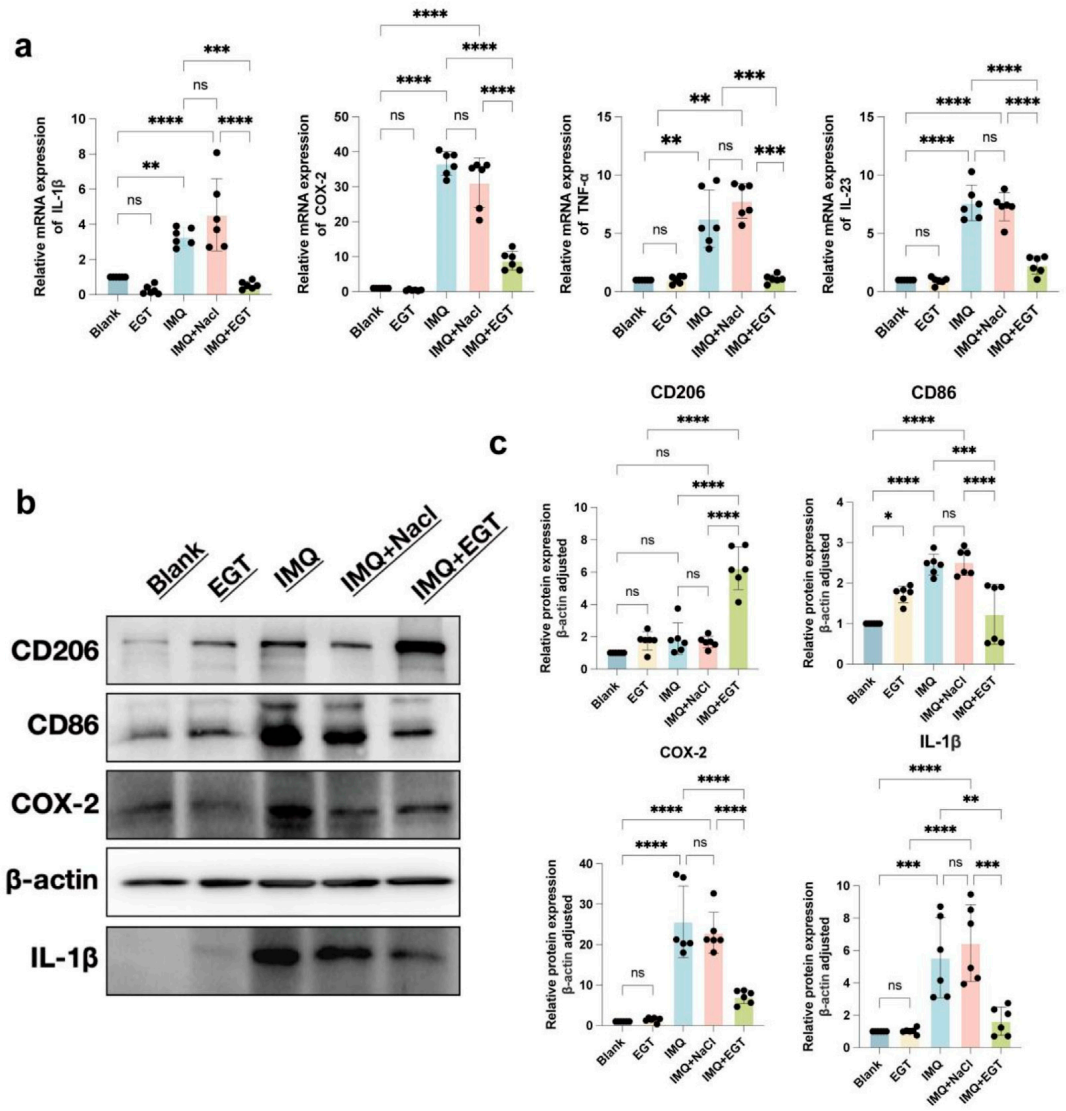
Ergothioneine attenuates IMQ-induced psoriasis-like skin symptoms. **(A)** COX-2, IL-23, IL-1β and TNF-α mRNA expression on the back of psoriatic mice. **(B, C)** Protein expression of skin inflammatory factors on the back of psoriatic mice and statistical graphs. Data are expressed as mean ± SD. *p < 0.05; p < 0.001; p < 0.0001.

To further assess the inflammatory state of the mice, we performed pro-inflammatory cytokine expression assays at the mRNA and protein levels in imiquimod-treated mice. The results showed that the expression levels of COX-2, IL-23, IL-1β and TNF-α genes were significantly increased in the dorsal skin of mice in the IMQ model group. Compared to the model group, the addition of EGT significantly reduced the expression of the above pro-inflammatory factors (Figures 5A–C). In addition, immunohistochemical results showed that EGT significantly reduced the number of iNOS-positive cells in skin tissues compared to the IMQ group (Figures 4E, F). Of particular interest, the introduction of EGT also led to a significant decrease in the protein expression of the M1 macrophage-specific marker CD86 and a corresponding increase in the expression of the M2 macrophage marker CD206 (Figures 4G–J). This finding suggests that EGT may have the potential ability to regulate the macrophage M1/M2 ratio and the body’s immune response, which could have a positive impact on the treatment of autoimmune diseases such as psoriasis.

### EGT inhibited M1 macrophages and increased M2 macrophage infiltration in mice with psoriasis

**FIGURE 6:**
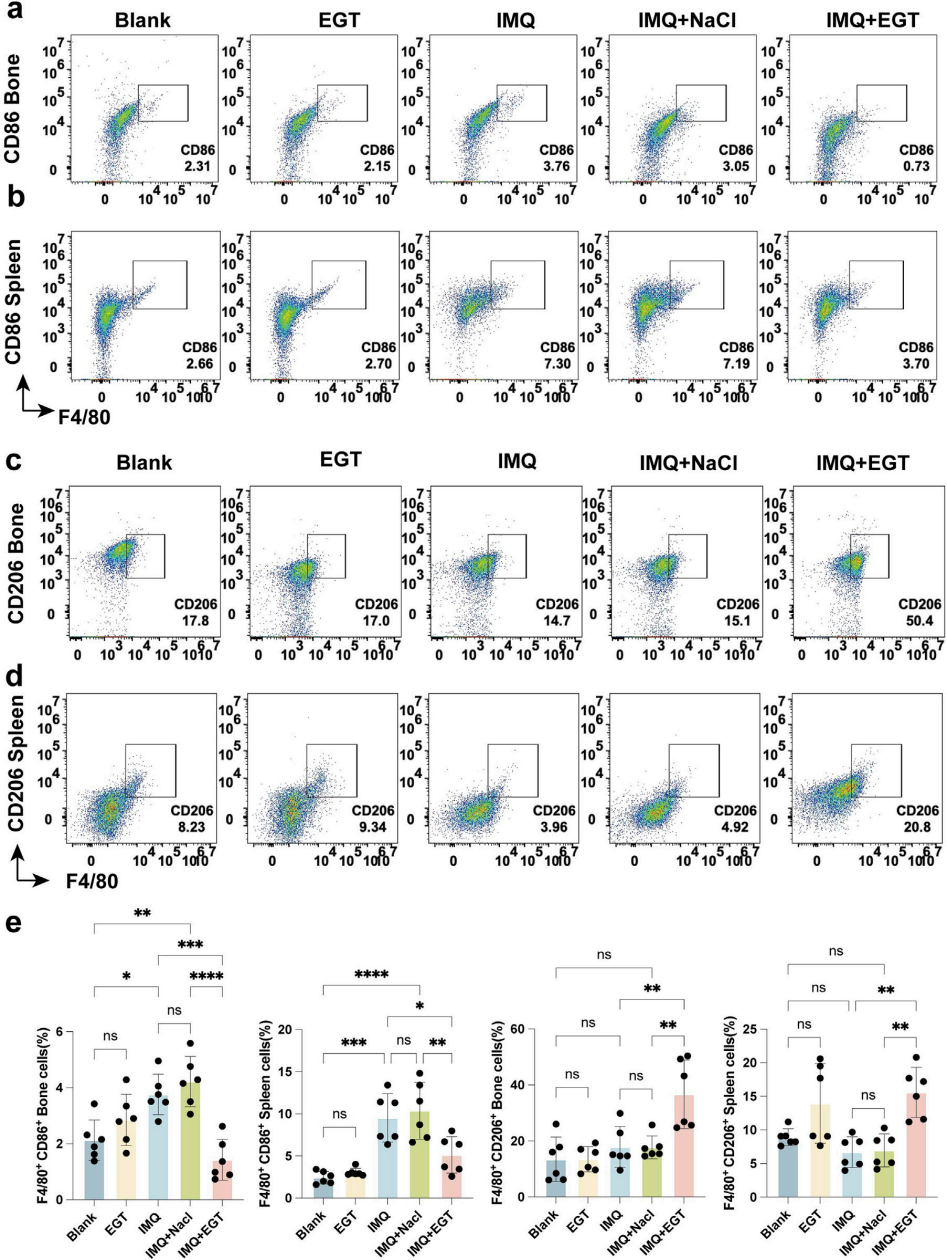
Ergothioneine alters the M1/M2 macrophage ratio in the immune microenvironment of psoriatic mice. **(A, B)** Percentage of CD86 F4/80 M1 macrophages in bone marrow and spleen of mice with psoriasis detected by flow cytometry. **(C, D)** Percentage of CD206 F4/80 M1 macrophages in bone marrow and spleen of psoriatic mice by flow cytometry. **(E)** Statistics of the percentage of M1/M2 macrophages in the spleen and bone marrow. Data are SD ± mean. *p < 0.05; p < 0.001; p < 0.0001.

In the above study, we clearly observed a significant negative correlation between the concentration of EGT and the ratio of macrophage M1/M2 in mice. To further investigate this relationship, we examined the specific changes in macrophage levels in a psoriasis-like dermatitis model induced by imiquimod. Using immunohistochemical staining techniques, we found that topical application of imiquimod significantly increased the number of CD86-positive cells within the dorsal skin lesion area, with a corresponding decrease in the number of CD206-positive cells (Figures 4G–J). In addition, in-depth analyses using flow cytometric analysis techniques further revealed the specific effects of EGT on macrophage subtypes. The results of the study showed that no significant effect on mouse macrophages was observed in the case of EGT application alone. However, the number of M1 macrophages (F4/80 CD86) infiltrating the skin of lesions in the model group was significantly higher than in the control group (Figures 6A, B). Notably, M2 macrophages (F4/80 CD206) did not show significant quantitative changes between the control and model groups. When EGT was applied to the mice, we observed a significant decrease in the percentage of F4/80 CD86-labelled M1 macrophages and a significant increase in the percentage of F4/80 CD206-labelled M2 macrophages in the spleen and bone marrow of the mice (Figures 6C, D). This finding suggests that EGT may modulate the secretion of pro-inflammatory cytokines by inhibiting the infiltration of M1 macrophages and enhancing the anti-inflammatory effects of M2 macrophages to achieve therapeutic efficacy in psoriasis (Figure 6E).

### Investigation of the mechanism of action of EGT in the treatment of psoriasis using network pharmacology

**FIGURE 7:**
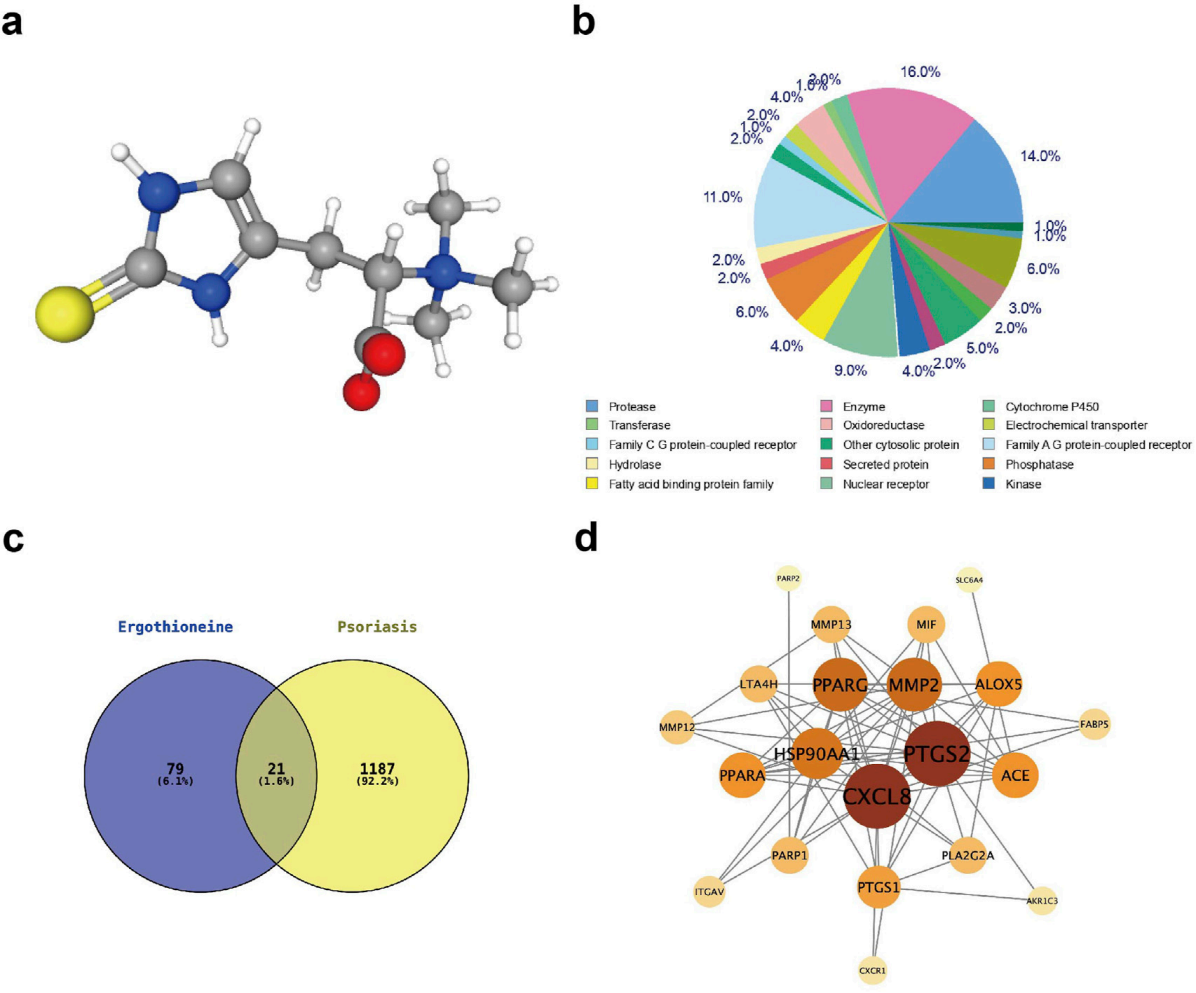
Network pharmacological analysis of EGT to investigate the site of action of EGT in psoriasis. **(A)** The chemical structure of EGT was examined using the PubChem database, with yellow spheres representing sulphur, grey spheres representing carbon, blue spheres representing nitrogen, red spheres representing oxygen and white spheres representing hydrogen. **(B)** Analysis of the potential EGT target gene type representation using the SwissTargetPrdiction database. **(C)** Intersection of predicted EGT target genes and psoriasis-causing genes analysed using Vernon analysis, resulting in 21 genes with common effects. **(D)** A protein-methionine interaction network was constructed using the String database for the 21 target genes screened for colocalisation.

The chemical structure of EGT was analysed using the PubChem platform (http://pubchem.ncbi.nlm.nih.gov/). Specifically, the three-dimensional chemical structure of EGT (Figure 7A), in which sulfur atoms are shown as yellow spheres, carbon atoms are shown as grey spheres, nitrogen atoms are shown as blue spheres, oxygen atoms are shown as red spheres, and hydrogen atoms are shown as grey spheres. To further investigate the biological effects of EGT, we explored the target genes interacting with EGT using the swissTarget prediction database and counted the major categories of EGT target genes (Figure 7B), which showed that enzymes (accounting for 14.0% of the total), proteases, and other enzymes and proteins were the most abundant categories among them. This finding provides an important direction for further studies into the mechanism of action of EGT and its biological functions. In addition, this study used the GeneCards database to screen for causative genes associated with psoriasis. Using intersection analysis, we predicted and identified 21 genes that are both causative genes of psoriasis and potential target genes of EGT (Figure 7C). To further elucidate the mechanism of these common target genes in the pathogenesis of psoriasis, we selected 12 of these key target genes and constructed a protein-protein interaction network using the String database. The Degree algorithm in CytoScape software was then used to rank the importance of these common genes, and the results showed that genes such as PTGS2 (COX-2) and CXCL8 played the role of core target genes in the process of ergothioneine in alleviating psoriasis (Figure 7D).

### EGT exerts anti-psoriasis effects through inhibition of NF-κB/JAK-STAT3 signalling

**FIGURE 8:**
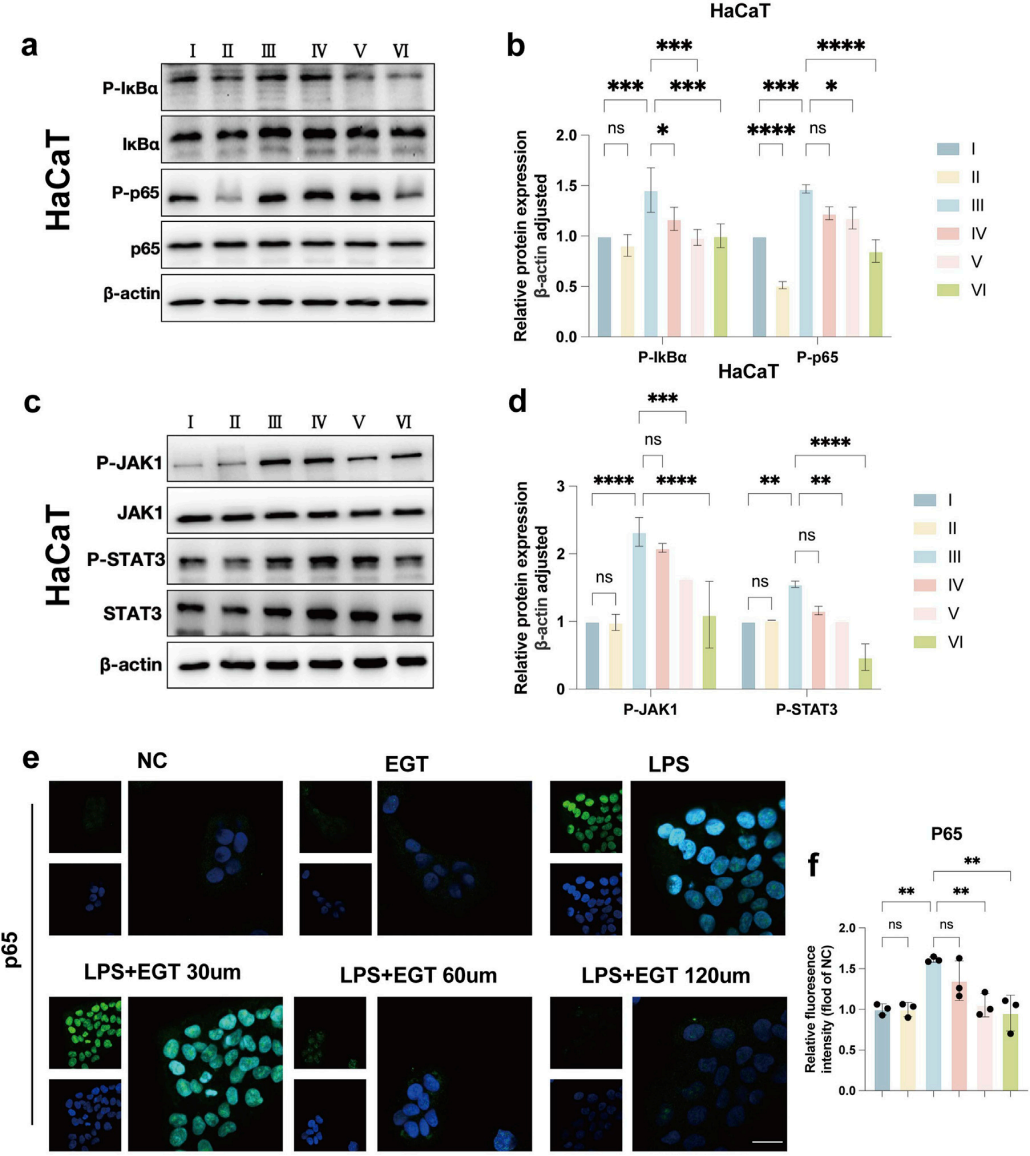
EGT exerts anti-psoriasis effects by inhibiting NF-κB/JAK-STAT3 signalling. **(A)** Western blot detection of IKB-α and NF-κB p65 and their phosphorylation levels in HacaT cells stimulated with LPS and treated with EGT. **(B)** Quantification of protein levels in **(A)** using grey value analysis with ImageJ software. **(C, D)** Western blot detection of JAK1 and STAT3 and their phosphorylation levels after treatment of HacaT cells with EGT stimulated by LPS, and protein levels were quantified. **(E, F)** Immunofluorescence staining results of NF-κB p65 in each group after treatment of RAW264.7. Data are SD ± mean. *p < 0.05; p < 0.001; p < 0.0001.

Through network pharmacology studies, we identified a potential mechanism of action of EGT in anti-psoriasis. To further validate this finding, we verified the effect of EGT on the cellular effects of the NF-κB/JAK-STAT3 pathway at the *in vitro* level. The experimental results were as expected and clearly showed that EGT effectively inhibited the phosphorylation process of IKBα, NF-κB p65 and JAK1, STAT3 in HaCaT cells (Figures 8A–D). In addition, repeated experiments performed on RAW264.7 cells led to the same conclusion (Supplementary Figures S4). By immunofluorescence analysis, we observed that the LPS-induced nuclear translocation of p65 was significantly reduced by the addition of EGT (Figures 8E, F). The above series of experimental results confirmed the inhibitory effect of EGT on the NF-κB pathway. Therefore, we believe that the preventive effect of EGT on psoriasiform dermatitis may be realized through the inhibition of NF-κB/JAK-STAT3 mediated inflammatory response.

## Disscusion

Psoriasis is a chronic autoimmune-mediated dermatological condition characterised by inflammatory processes and the overproduction of cytokines. Impairment of the skin barrier function is also a key feature of the disease (Lowes et al., 2014). It follows that effective therapeutic strategies for this condition should include the modulation of skin barrier function and the reduction of skin inflammatory responses. Nevertheless, the potential adverse effects and high costs associated with the long-term use of biologics and synthetic drugs represent a significant challenge to the current treatment paradigm (Tremblay and Bissonnette, 2002; Rizvi et al., 2015). Consequently, the pharmaceutical industry is engaged in the active pursuit of novel, safer, and more efficacious pharmacological agents for the treatment of psoriasis (Krueger and Duvic, 1994; Russo et al., 2004; Zhang et al., 2024). The current pharmacological treatments for psoriasis act through a variety of mechanisms, including, but not limited to, the inhibition of keratinocyte hyperproliferation, the restoration of normal cellular differentiation, the inhibition of multiple inflammatory signalling pathways (e.g., NF-κB, JAK-STAT3), and the attenuation of the inflammatory response (Li et al., 2023). Furthermore, a substantial body of experimental evidence has emerged indicating that the identification of macrophage activation states and the modulation of their polarization direction can facilitate the diagnosis and treatment of select immune-associated inflammatory diseases. It is our hope that the present study will succeed in altering the M1/M2 macrophage ratio in the psoriatic skin immune microenvironment by modulating macrophage polarisation in psoriatic dermatitis. Furthermore, we aim to induce macrophage differentiation towards M2 or M2-like differentiation and neutralise the over-excited pro-inflammatory response in the skin, thereby achieving the therapeutic goal of psoriasis (Bell et al., 2003; Baker et al., 2003; Ko and Liao, 2023).

In recent years, EGT has attracted much attention due to its significant anti-inflammatory and antioxidant effects and extensive safety record in humans (Xiong et al., 2024). However, there is a lack of definitive evidence regarding the specific protective role of EGT in the development of psoriasis. The present study investigated the potential of EGT in the treatment of psoriasis and found that its anti-inflammatory effects significantly inhibited disease progression in an IMQ-induced model of psoriasis. The results of *in vivo* experiments showed that EGT was able to significantly reduce pathological changes such as hyperkeratosis, keratinisation, epidermal thickening and inflammatory cell infiltration in dorsal skin samples, and effectively ameliorated the systemic side effects of psoriasis and the levels of inflammatory mediators in skin tissues in mice. Meanwhile, *in vitro* experiments also showed that EGT was able to downregulate the expression of CD86, an M1-type marker, and upregulate the expression of CD206, an M2-type marker, in LPS-stimulated mouse macrophages, thereby regulating macrophage polarisation status. In addition, EGT was able to attenuate the inflammatory response of HaCaT cells under LPS stimulation and significantly reduce the phosphorylation levels of IκBα, NF-κB p65 and JAK1, STAT3.

In conclusion, the results of this study are consistent in indicating that EGT has a significant inhibitory effect in psoriasis. However, there are still some limitations in this study, and the specific mechanism by which EGT inhibits NF-κB/JAK-STAT3 to exert anti-inflammatory effects needs to be further investigated. In addition, the use of imiquimod to induce psoriasis *in vitro* in this study is relatively non-specific, and we expect to use K14-VEGF and K5-STST3C transgenic mouse models to further validate the therapeutic effect of EGT in psoriasis in the future.

## Data Availability

The original contributions presented in the study are included in the article/Supplementary Material, further inquiries can be directed to the corresponding author.
