# Supplementary material for: Ergothioneine attenuates psoriasis symptoms through modulation of M1/M2 macrophage polarisation via the NF-κB/JAK-STAT3 pathway
Source: Front Pharmacol. 2025 Feb 19;16:1521743. doi: 10.3389/fphar.2025.1521743 (PMC11880282; doi:10.3389/fphar.2025.1521743)
Supplement: Supplementary file 1 [file DataSheet1.docx]

**Ergothioneine attenuates psoriasis symptoms through modulation of M1/M2 macrophage polarisation via the NF-κB pathway**

Ang Li^1&^，Yanjie Liu^2&^, Peiling Yu^3&,^ Zhiyuan Zhang^4^,Tengxiao Huang^5^, Hang Li^5^, Songzhi Wu^1^, Xiaoyu Rong^1^, Wensheng Liao^1^, Hongqiang Wang^1^, Yanzheng Gao^1*^

&: These authors contributed equally to this work.

* : Corresponding authors

1: Department of Orthopedics, Henan Provincial People’s Hospital, People's Hospital of Zhengzhou University , Zhengzhou, Henan, 450003, China

2: Department of Rehabilitation, Henan Second Provincial People’s Hospital, Xinzheng, Henan, 451191, China

3: Department of Pathology, School of Basic Medical Sciences, Shandong University, Jinan, Shandong, 250012, China.

4: Department of Pathology, Qilu Hospital, Cheeloo College of Medicine, Shandong University, Jinan, Shandong, 250012, China.

5: Department of Orthopedics, Qilu Hospital, Cheeloo College of Medicine, Shandong University, Jinan, Shandong, 250012, China.

**Contact Information：**

Gao Yanzheng: Department of Orthopedics, Henan Provincial People’s Hospital, People’s Hospital of Zhengzhou University, No.7 Weiwu Road, Jinshui District, Zhengzhou, 450003, Henan Province, China (E-mail: yanzhenggaohn@163.com).

**Supplementary Table 1. Primer sequence in the study**

| **Gene** | **Primer** | **Sequence (5′-3′)** |
| --- | --- | --- |
| TNF-α（Mus) | Forward Primer  Reverse Primer | CCAACATGCTGATTGATGACAC  GAGAATGCCAATTTTGATTGCCA |
| IL-1β（Mus) | Forward Primer  Reverse Primer | TTCAGGCAGGCAGTATCACTC  GAAGGTCCACGGGAAAGACAC |
| COX-2（Mus) | Forward Primer  Reverse Primer | CATCCAACGTGACCCAGTGTT  AAATGCGTTCAGGACCGTCTT |
| IL-23（Mus) | Forward Primer  Reverse Primer | CCAGCGGGACATATGAATCTACT  CCTTGAGTCCTTGTGGGTCA |
| β-Actin（Mus) | Forward Primer  Reverse Primer | ACCTTCTACAATGAGCTGCGT  TACATGGCTGGGGTGTTGAAG |
| TNF-α（Homo) | Forward Primer  Reverse Primer | GAGGCCAAGCCCTGGTATG  CGGGCCGATTGATCTCAGC |
| IL-1β（Homo) | Forward Primer  Reverse Primer | TGATGGCTTATTACAGTGGCAA  GTCGGAGATTCGTAGCTGGA |
| IL-6（Homo) | Forward Primer  Reverse Primer | CAATGAGGAGACTTGCCTGGT  GCAGGAACTGGATCAGGACT |
| β-Actin（Homo) | Forward Primer  Reverse Primer | CATGTACGTTGCTATCCAGGC  CTCCTTAATGTCACGCACGAT |

**Supplementary Table 2. Antibody list in the study**

| **Antibody** | **Company** | **Catalog No.** | **Assay** | **Dilution** |
| --- | --- | --- | --- | --- |
| Anti-CD206 | Abacm UK | ab300621 | WB  IHC  IF | 1:1000  1:2000  1:500 |
| Anti-NF-κB P65 | Abacm UK | ab32536 | WB  IF | 1:1000  1:50 |
| Anti-Phospho-NF-κB p65 | Cell Signalling  USA | Ser536 #3033 | WB | 1:1000 |
| Anti-CD86 | Cell Signalling  USA  Abcam, UK | E5W6H#19589  E5W6H#19589  ab239075 | WB  IHC  IF | 1:1000  1:50  1:100 |
| Anti-IL-1β | Abways China | CY5087 | WB | 1:1000 |
| Anti-COX-2 | Abways China | CY8852 | WB | 1:1000 |
| Anti-β-actin | Abways China | AB0035 | WB | 1:1000 |
| Anti-iNOS | Abways China  Abcam, UK  Abcam, UK | CY5993  ab283655  ab283655 | WB  IHC  IF | 1:1000  1:2000  1:250 |
| Goat Anti-Rabbit IgG(H+L) HRP | Abways. China | AB0101 | WB | 1:5000 |
| FITC anti-F4/80 | eBioscience China | 11-4801-85 | Flow | 1:2000 |
| PE anti-CD86 | eBioscience China | 12-0862-82 | Flow | 1:100000 |
| APC anti-CD206 | eBioscience China | 17-2061-82 | Flow | 1:100000 |
| Anti-JAK1 | Cell Signalling  USA | #3332 | WB | 1:1000 |
| Anti-Phospho-JAK1 | Cell Signalling  USA | #3331 | WB | 1:1000 |
| Anti-STAT3 | Cell Signalling  USA | #9139 | WB | 1:1000 |
| Anti-Phospho-STAT3 | Cell Signalling  USA | #9145 | WB | 1:1000 |


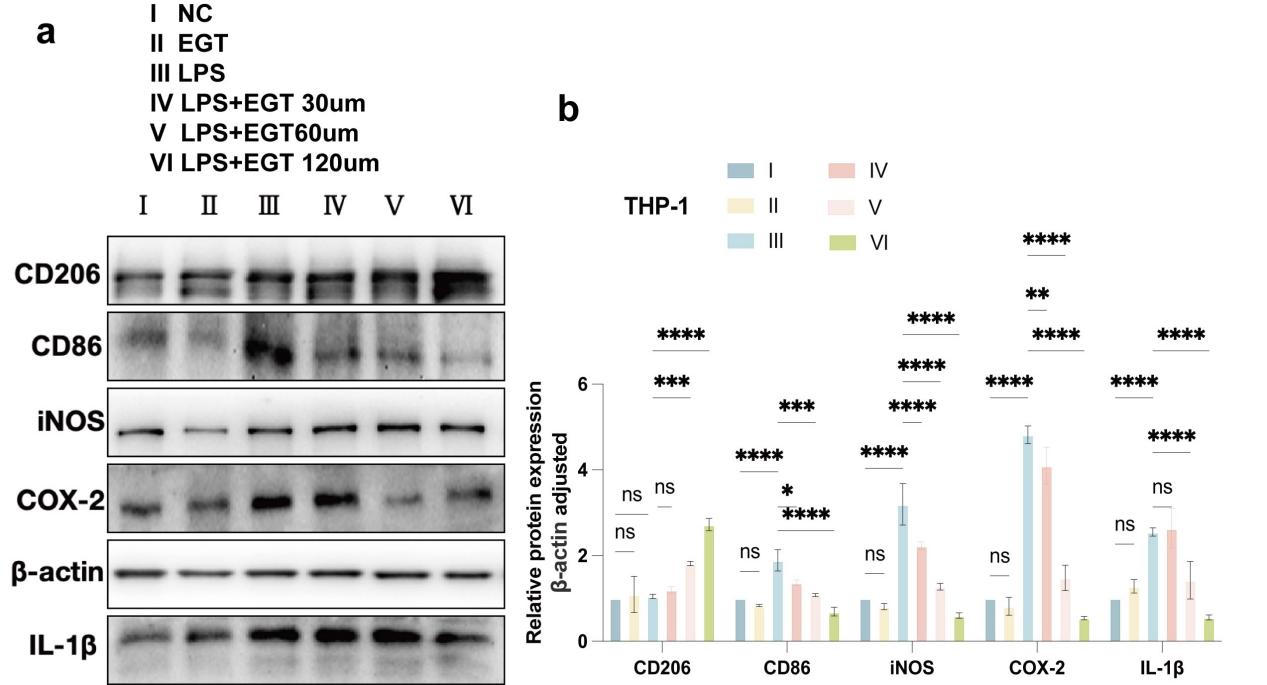


**Supplementary Fig1. EGT reduced the ratio of intracellular M1/M2 macrophages and the expression of pro-inflammatory factors after LPS induction in THP-1 cells. (a) THP-1 cells were treated with LPS in the presence or absence of EGT for 24 hours. Protein expression of pro-inflammatory factors in each group was then quantified. (b) A statistical graph of protein quantification in (a) was generated. n=3. Data are expressed as mean ±SD . *p < 0.05; p < 0.001; p < 0.0001.**


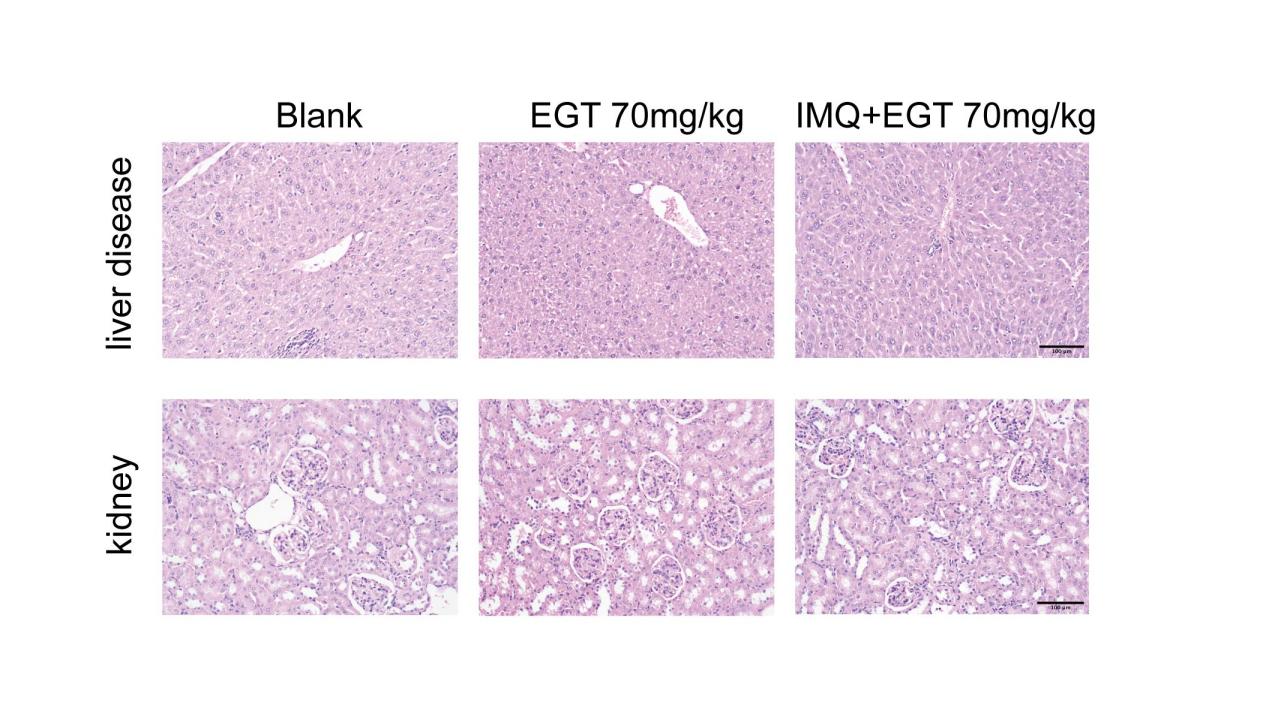


Supplementary Fig2. Histopathologic HE results of mouse spleen and kidney.


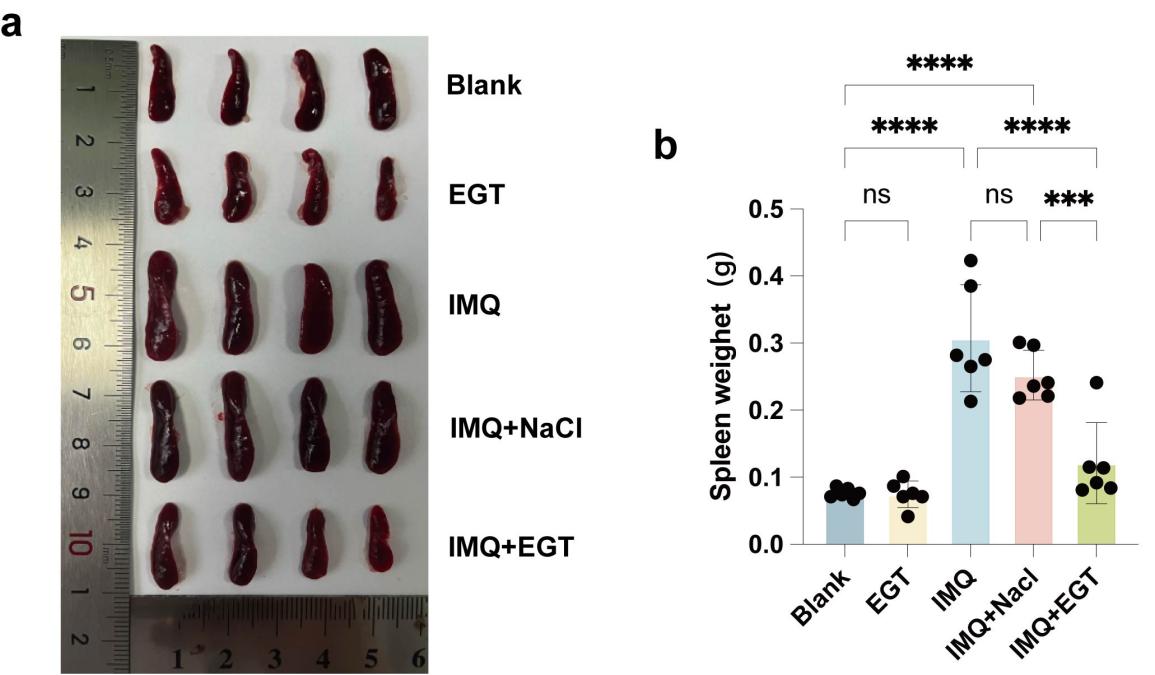


**Supplementary Fig3. The introduction of EGT has been observed to alleviate the symptoms of psoriasis in mice.** (a) Photographic documentation of the spleens of psoriasis model mice in each experimental group on day 7 of the study. (b) A statistical representation of the spleen weight of mice. Data are expressed as mean ±SD . *p < 0.05; p < 0.001; p < 0.0001.


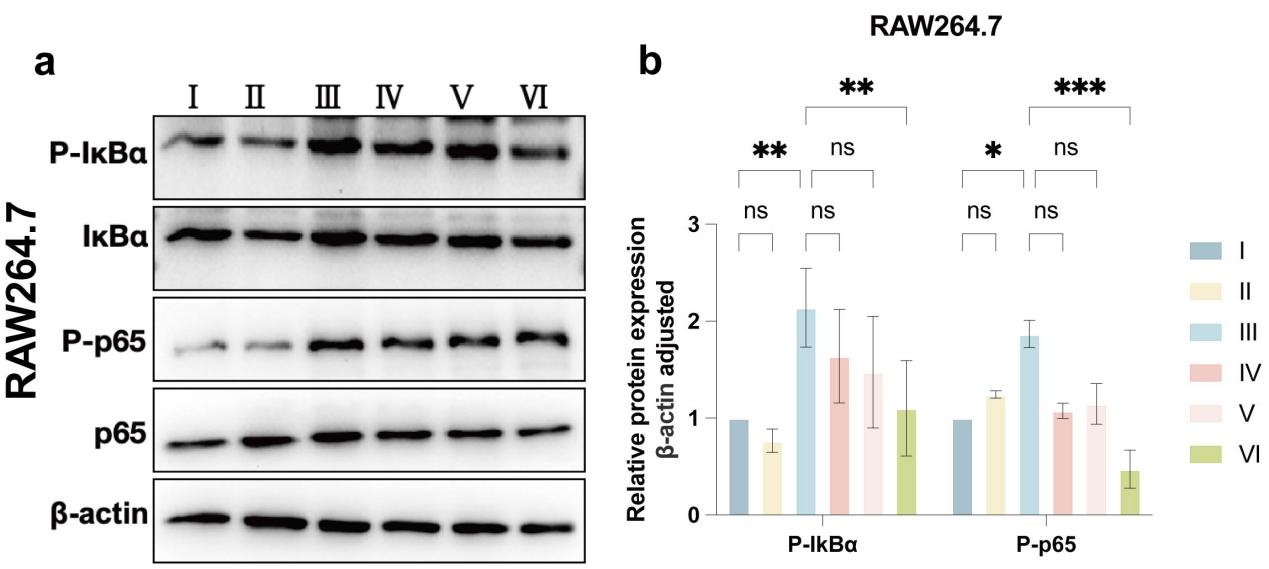


**Supplementary Fig4.** **EGT exerts anti-psoriasis effects by inhibiting NF-κB signaling.**

(a)Western blot detection of IKB-α and NF-κB p65 and their phosphorylation levels in RAW264.7 cells stimulated with LPS and treated with EGT. (b) Quantification of protein levels in (a) using grey value analysis with Image J software. n=3. Data are expressed as mean ±SD . *p < 0.05; p < 0.001; p < 0.0001.
